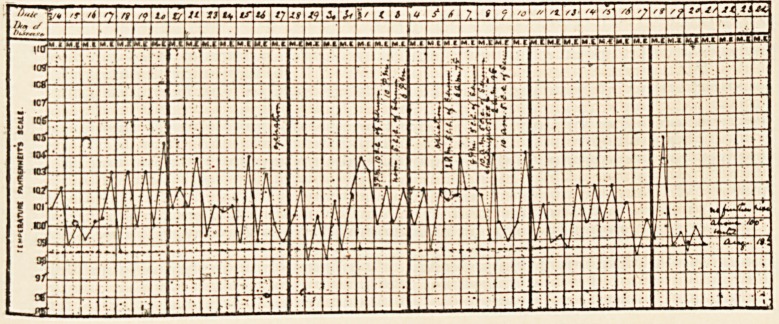# Cases of Hepatic and Intestinal Surgery

**Published:** 1897-12

**Authors:** Charles A. Morton

**Affiliations:** Professor of Surgery in University College, Bristol; Surgeon to the Bristol General Hospital


### CASES OF HEPATIC AND INTESTINAL SURGERY.

BY

CHARLES A. MORTON, F.R.C.S. Eng.,

*Professor of Surgery in University College, Bristol; Surgeon to the Bristol General Hospital.*

THE following cases, which have been under my care during the past summer, seem to me to form such an interesting group that I have felt inclined to publish them as such:

### Large Gallstone floating in greatly dilated common duct—High temperature with rigors—Removal of stone, with primary union of wound in duct.

S. C., aged 72, was seen by me in consultation with Dr. Michell Clarke on May 16th, 1897. She had been admitted into the General Hospital under Dr. Clarke's care on May 3rd. For eight months before admission she had been subject to attacks of pain in the right hypochondriac region, and vomiting, and these attacks had been much worse for some weeks before she came into the hospital. Directly after admission the vomiting ceased, but at first every day, and then every few days, she got attacks of pain in the same situation, lasting for some hours. Her temperature is shown on the chart. On the 7th and 14th she had definite rigors. She had been an in-patient nine years before with apparently similar attacks. There was decided jaundice, but no swelling in the position of the gall-bladder, though tenderness was very marked in that situation, and not elsewhere in the abdomen.

It seemed to us clear that she was suffering from gallstones, but the rise of temperature night after night seemed more suggestive of suppurative cholangitis than of the intermittent fever described by Charcot, Osler, and others as occasionally present in cases in which a gallstone lies in the common duct and has not set up suppuration in the gall tracts. Although the patient had attained a considerable age, we felt that operation gave her the best chance of recovery.

I operated on May 17th. On opening the abdomen I could just feel the fundus of the gall-bladder, and could make out the presence of a calculus within it, but it was intimately adherent to the colon. I partly cut and partly tore through these adhesions and others to the duodenum, and between this portion of bowel and the colon, and thus exposed the gall-bladder, which appeared shrunken and nearly empty. On its left side, and adherent to it, was a sausage-shaped fluid swelling, lying in the position of the common duct. In the lower portion of this a very large stone could be felt, which readily floated into the upper end of the swelling when pressed upon. This sausage-shaped swelling was evidently a greatly dilated common duct. It was between two and three inches in length and an inch in diameter. The walls did not feel thick.

I thoroughly shut off the little fossa under the liver, in which the gall-bladder and dilated duct lay, from the rest of the peritoneal cavity with sponges, and then aspirated both the gall-bladder and the dilated common duct, and, having incised them, removed the stone contained in each. The one in the common duct was the size of a small walnut (nearly three inches in circumference), and the one in the gall-bladder was the shape of a conical bullet and a little more than an inch in length. On passing my finger into the dilated common duct, I found it contracted to an apparent cul-de-sac below and to a small tube above. During the extraction of the stone and suturing of the incision in the duct (an inch in length) clear watery bile kept oozing out, and had to be constantly sponged up. The large stone was quite easily pressed out of the incision in the duct. The incisions in both the common duct and the gall-bladder were united with fine silk sutures, and the fossa drained with an India-rubber tube and iodoform gauze packing. The abdominal wound was partly closed around this drain. There was no shock from the operation. During the first forty-eight hours there was just a trace of bile discharged from the tube. The gauze was removed at the end of the first forty-eight hours, and the tube on May 22nd; and by the end of the month the wound had closed. There was no return of pain or pyrexia after the operation, and the jaundice gradually passed off. She is now (October, 1897) in very good health.

The condition of the temperature in this case is of great interest. It was not of the intermittent type which we find now and then associated with ball-valve obstruction of the common duct by a gallstone, but the nightly rise to 102°—104° seemed to indicate that suppuration had been set up in the gall tracts. Suppurative cholangitis was not however present, and the temperature was undoubtedly due to the presence of the large gallstone floating up and down in the enormously dilated common duct. The success of the choledochotomy in a patient 72 years of age is encouraging, and shows the advantage of operations for gallstones in the aged. With so large a stone, no other method than incision of the duct would commend itself to an operator; but the rapid healing of the wound in the duct seems to me to indicate the advantage of incision and suture of the duct over such methods as needling and crushing, which are always attended by a certain amount of risk of injury to the duct, or of failure in the passage of the fragments.

**Mucous cyst in the interior of the cæcum and connected with the base of the appendix—Excision of the cyst with appendix and surrounding cæcal wall—Primary union of wound in cæcum.**

T. F., aged 39, was sent to me at the Bristol General Hospital by Mr. Boyce, of Wotton-under-Edge, on June 23rd, 1897. His illness began three months before, with pain in the right side of the abdomen, and this pain had recurred at intervals of a few days up to the time I saw him. It had always been limited to the right side of the abdomen, and now and then localised just above McBurney's spot. When the pain had been severe, there had often been vomiting. The bowels had been confined, and he had had to take purgatives frequently. He had only once noticed any abdominal distension, and this was after a severe attack of pain, unaccompanied by vomiting. He had lost 37 lbs. in weight, for he had been unable to take solid food, as it brought on the attacks of pain. He first noticed a lump in his abdomen a fortnight before admission. It had never altered its position, but had been sometimes more prominent than at others.

Just above McBurney's spot, in the position of the ascending colon, was a firm, deep swelling, the size of a small hen's egg, which rolled under the fingers from side to side, but could not be displaced for any distance. The signs and symptoms seemed to point distinctly to some form of growth in the wall of the colon; and as carcinoma is the only form of growth which is common in this situation, there seemed little doubt it was of this nature, and I intended to excise the affected portion of bowel and unite the divided ends. It did not seem fixed enough for a swelling (of that size) due to disease of the appendix.

I operated on June 26th. When I introduced my finger into the abdomen I could readily feel the swelling as a smooth, round mass, moving about freely within a limited range; and on further examination I discovered that it lay within the cæcum, and was tense but fluctuating.

I found the appendix much thickened and adherent near its base to the cæcum, and withdrew it and part of the cæcum through the incision in the abdominal wall. Having isolated the cæcum with iodoform gauze packing within the abdomen, and surrounded the protruding portion with a carbolised towel, I separated the adhesions between the base of the appendix and the cæcum, and then opened the cæcum over the tumour along the longitudinal band which runs to the base of the appendix, and found a thick-walled cyst continuous with the wall of the cæcum at its junction with the appendix. I then cut all round the attachment of the cyst and appendix to the cæcum, and removed them together. This left a large gap in the cæcum, but only a little mucus escaped. This gap was closed with fine silk sutures. After carefully sponging with 1 in 2000 biniodide of mercury solution the iodoform gauze packing was removed, the portion of cæcum sutured was returned into the abdomen, and Morris's wick-drain inserted over it, and around this the incision in the abdominal wall was sutured layer by layer. This drain was removed six hours later. There was no shock from the operation. The patient was fed entirely by rectal injections (as he was a strong man) until the 28th, and then he began to take liquids by the mouth. After removal of the gauze drain the wound at once healed; and he was discharged quite well, and with an increase of weight, on July 23rd.

The nature of the cystic swelling in the cæcum is of great interest. I cannot find a description of any similar growth. That it was not the dilated end of an obstructed appendix will be clear from the description which follows. Doubtless the growth of the cyst had obstructed the lumen of the appendix, and led to the cystic dilatation which was present to a slight extent at the tip of the appendix.

The cyst projected $1\frac{1}{2}$ inches into the cæcum. The surface was smooth and of a deep red colour. The mucous membrane of the cæcum terminated in a ridge around its base, and was clearly not continuous with it. The cyst was partly buried in the cæcal wall. Its wall was about $\frac{1}{8}$ of an inch in thickness, and in appearance resembled a thickened layer of muscle in the intestinal wall. It was full of amber-coloured jelly, which was so adherent that it had to be washed and scraped out. A very fine probe (not an ordinary probe) could just be passed from the appendix into the cyst, but the wall of the cyst was seen to be quite distinct from that of the cæcum or appendix on careful dissection. The interior of the cyst was smooth and slate-coloured, and resembled mucous membrane in appearance; and on it were numerous chalky-looking patches, some of which were quite soft and others were calcareous. Microscopic sections do not show any mucous membrane either covering or lining the cyst. The cyst-wall is seen to be composed of fibrous tissue only, and to be clearly differentiated from the muscle-fibre of the cæcal wall.

The muscular wall of the appendix was much hypertrophied, and the mucous membrane seemed also thickened; but the lumen was large except near the tip, where it was contracted, though still admitting a fine probe, and beyond there was a small collection of watery fluid in the dilated extremity of the tube. The specimen shows that complete obstruction of the cæcal end of the appendix does not necessarily lead to great distension with mucus.

**Strangulated umbilical hernia—Gangrene of nearly a foot of transverse colon—Resection of gangrenous bowel and immediate union with Murphy's button—Recovery.**

H. H., aged 59, was admitted into the General Hospital late in the evening of June 1st, 1897. She had been ruptured for 29 years, but had always worn an abdominal belt. At 10 o'clock on June 1st, the rupture doubled its ordinary size, and became very painful, and she vomited constantly up to the time of her admission the same evening. I saw her soon after midnight, and found an extremely tense, resonant umbilical hernia, the size of a slop-basin, without impulse on coughing, and with red œdematous skin over it. The pain was in the rupture. Her pulse was 100, and her general condition seemed very good.

I operated immediately. On opening the sac I found a layer of omentum spread over the bowel, and on turning this aside came on bowel which was black, and smelt like a decomposing corpse. Nearly a foot of transverse colon was discovered in this condition, and there was a large mass of nearly black omentum. The constriction of the ring was extremely tight. After thoroughly irrigating the gangrenous bowel with biniodide of mercury solution, I exposed first the upper and then the lower edge of the ring, and divided them with blunt-pointed scissors; and then drew out more bowel, so as to get beyond the gangrenous area, but not more than a few inches at each end of the gangrenous portion could be withdrawn. To each of these portions I applied two Arbuthnot Lane's clamps, and then resected the gangrenous part and united the divided ends with Murphy's 1-inch button. The proximal portion of colon was rather dilated, and was thick from œdema, so that it was rather difficult to get the ring of bowel well within the grasp of the button. No fæces escaped during the operation. I cut off a handful of gangrenous omentum, and then, after dividing some adhesions about the neck of the sac, returned the stump of omentum and the united end of colon within the ring. The centre of the sac was then cut away, and the margins stitched together over the ring, with an iodoform gauze drain tucked against the united colon and India-rubber tubes inserted into the lower angle of the wound. There was no shock. Pulse was 120 during the operation, and not more than 108 at the end, and was very good.

A week after the operation there was some fæcal leaking from the wound, and for some days this was very free; but as soon as the bowels began to act freely *per anum*, it lessened, and by the end of the month had become very slight. The button was passed on July 2nd, *i.e.* a month and a few days after the operation. She was discharged at the end of July, with the fistula almost closed; and when seen on August 17th, there was only a slight occasional stain from a very minute opening. This closed soon after.

The rapidity with which gangrene occurred in this case is very remarkable. It is well known that in inguinal or femoral hernia, when the bowel is very tightly gripped, it may become gangrenous in a few hours; but so rapid an onset of gangrene must be very rare in an umbilical hernia. The remarkably good general condition of the patient with such extensive gangrene is interesting. There was none of that collapse, with cessation of pain in the hernia, so often described as a sign of gangrene.

The case seems to me also to illustrate the advantage of immediate union by Murphy's button in such a condition. I am quite sure it does not take longer to unite the bowel with the button than it would to carefully suture the ends to the margins of the incision into the sac. Fæcal extravasation at the time of operation is avoided; and even if leakage of fæcal matter subsequently occurs, the peritoneal cavity will be safely shut off by adhesions, and only a fæcal fistula, not an artificial anus, will form, and will probably close spontaneously. A drain down to the united portion of bowel obviates the danger of extravasation of fæcal material into the peritoneal cavity, should leakage occur. On the other hand, it may be urged that if the adhesions present at the neck of the sac before operation are left undisturbed, the safety of the general peritoneal cavity is best secured. But it has been shown that the safety of the patient from peritoneal extravasation is not always secured by these adhesions about the neck of the sac, for in several cases the bowel has given way on the abdominal side of these adhesions, and set up fatal peritonitis. Moreover, the mechanical obstruction is not always relieved until the neck of the sac is divided, Hence, even if we do not unite the bowel with Murphy's button, we must at least separate the adhesions at the neck of the sac, and bring down healthy bowel; and it would certainly be a more rapid process to unite with Murphy's button, than to carefully stitch the ends (after resection of the gangrenous portion) to the skin. The time occupied by careful suturing of the divided ends together was the great objection to uniting them, but the use of Murphy's button has made union the shorter method. Space does not permit a full discussion of this very interesting question.[1]

### Pyæmic abscess of the liver due to suppurative appendicitis—Removal of appendix and drainage of liver abscess—Recovery.

A. W., aged 10, came under my care on May 27th, 1897. There was a history of an acute onset of vomiting, diarrhœa and abdominal pain, three weeks before I saw him. There had been pain in the right iliac region and over the liver, and his temperature had often been 103° or 104° at night. Some tenderness had been noticed in the right iliac region and over the left lobe of the liver during the second week of his illness; but a definite swelling had been observed in the iliac region only for two days. The boy looked pale, but not very ill. There was no great distension of the abdomen; but just below McBurney's spot was a firm, well-defined, sausage-shaped swelling. The left lobe of the liver was prominent and was distinctly tender. It seemed clearly a case of suppurative appendicitis, and from the pain and tenderness in the region of the liver, portal pyæmia was suspected. I operated at once, and found a collection of stinking pus shut in by adhesions between the appendix, cæcum, ileum, and omentum. There were no adhesions to the abdominal wall. A large quantity of serous fluid was present in the peritoneal cavity. I removed the appendix, and drained the peritoneum. He recovered well from the operation, and, with the exception of a little tenderness over the left lobe of the liver, had no symptoms until May 30th, when he had a rigor and tem-

[1] There have, of course, been several disasters after the use of Murphy's button, as there have after all methods of intestinal union. But the use of the button has now been established as one of the most satisfactory methods, and it has quite revolutionised the treatment of gangrenous hernia.

perature of 104°. There was then no increase of the tenderness over the liver, and no swelling in the region where the appendix trouble had been. The rigors recurred and the temperature frequently rose for the next few days, and the employment of antistreptococcic serum failed to have any beneficial effect. Distinct bulging and marked tenderness were present over the left lobe of the liver, and on June 4th I explored this portion of the liver by an abdominal incision. I found marked thickening of the liver capsule over the left lobe, but not to the right of the falciform ligament. There were no adhesions over the prominent left lobe. I passed the needle of the exploring syringe twice into the liver, with negative results; but on pushing it in the third time, to the depth of three inches, I got a drop of thick pus in the syringe. I then left the needle in and sewed the surface of the liver around to the edges of the incision in the abdominal wall, so as to shut off the general peritoneal cavity, and made a small incision through the thickened capsule by the side of the needle, and then worked along the needle track until a little pus escaped. At first the liver bled rather freely, and I had to make pressure until it ceased, and then bored on again to the depth of three inches. A small drainage tube was inserted, and iodoform gauze placed around it. There was no shock from the operation. On the day after the operation the dressing was found soaked with stinking pus. No doubt a large abscess deeply situated in the liver had ruptured into the track made to drain the smaller collection. On June 6th it was noted that the discharge from the liver abscess was still considerable, but was no longer offensive, and that the left lobe had become reduced in size; and by the end of July both the sinus from the liver abscess and from the region of the appendix had nearly closed, and he had gained flesh.

At the end of August he got return of pyrexia, and dulness developed in the splenic region and rapidly extended up to the angle of the scapula. The free use of an exploring needle under chloroform failed to discover pus, and, to my surprise and satisfaction, the pyrexia and swelling disappeared. Now (October) both sinuses are healed, and the boy seems well.

The result of the surgical treatment of pyæmic abscess of the liver is not usually successful, for there is usually more than one abscess of this nature, and these are not accessible for drainage. In this case evidently a large abscess was so nearly reached that it broke into the track made for the drainage of a much smaller collection. By the successful result we should, I think, be encouraged to operate even in pyæmic abscess of the liver.

This was not the first case of portal pyæmia from suppurative appendicitis which I have seen. In some cases there is an extra-peritoneal sub-diaphragmatic abscess in the region of the liver; but this condition is not due to portal pyæmia, but to extension of a collection of pus from the neighbourhood of the appendix upwards.

### Intussusception in infant aged three months—Reduction by operation—Recovery.

J. D. came under my care on July 4th, 1897, with the history that after the mother had retired to bed on the previous night the baby began to vomit and continued to do so at intervals all night, and at intervals a little blood was passed from the bowel. There was a firm elongated swelling lying midway between the umbilicus and the middle of Poupart's ligament. It did not vary in hardness from time to time. *Per anum*, I could feel it through the rectal wall, but I could not touch the apex of the intussusceptum.

At 2.30 p.m. chloroform was administered, and I allowed several ounces of water to pass into the bowel from a height of three feet, firm pressure being maintained around the enema nozzle at the anus, so that no fluid escaped until the abdomen was well distended. The swelling at once ascended on the left side of the abdomen to the epigastric region, and then disappeared altogether. When the fluid was allowed slowly to trickle out of the anus, the swelling could again be felt in the splenic region. I did not venture to inject more fluid in so young an infant for fear of rupturing the bowel, as the abdomen had been thoroughly distended with it. I thoroughly washed the abdomen and scrubbed it with 1 in 500 biniodide of mercury solution, and the baby was placed on a hot-water pillow and its limbs and chest wrapped in cotton wool. An incision was made in the upper part of the left semilunar line, *i.e.* near the swelling, and the latter was found to be the apex of an intussusception which filled the ascending and transverse colon. I easily and rapidly reduced it out of the transverse colon, but found great difficulty in kneading it out of the ascending colon, and more especially out of the cæcum, as my incision lay so much on the left side of the abdomen. However, at last I felt satisfied all swelling had disappeared out of the cæcal region, and I sutured the wound in three layers. After suturing the wound, another hard round lump appeared in the cæcal region, but with a little gentle pressure it disappeared. A pad of wool was firmly bandaged over the cæcal region.

There was no recurrence of the intussusception, and the infant was quite well by August 1st.

### General purulent peritonitis—Abdominal cavity irrigated and drained—Recovery.

P.S., aged 32, came under my care on May 16th, 1897, with the history that she had been suddenly taken ill five weeks before with severe pain in the abdomen, about the region of the umbilicus, and vomiting. The abdomen began to swell at once, and the pain and vomiting continued up to the time of her admission; she had lost much flesh, and for a week before admission had suffered from dysuria. The last child was 2½ years old; and menstruation had been regular up to a week before admission, when she missed a period.

The abdomen was greatly distended. Though nowhere quite dull, it was only resonant in the flanks. Below the umbilicus it was almost dull, and there was increased resistance but no definite swelling. The uterus was fixed and there was a tender mass lying behind it. She had a great deal of pain in the lower abdomen, and looked ill and worn. There was moderate pyrexia, and her pulse was rapid and weak.

On May 19th I opened the abdomen by a small incision midway between the umbilicus and the pubes and several pints of thick pus flowed out of the general peritoneal cavity. I found the omentum greatly thickened and vascular, adherent over the intestines and fixed to the peritoneum over the pubes, thus covering in all the pelvic organs. Small openings in this thickened omentum allowed the finger to be passed down into contact with adherent coils of bowel lying beneath. The parietal peritoneum was also greatly thickened, but no tubercles could be seen; neither did a section, subsequently made of a piece of thickened peritoneum removed at the time of operation, show any tubercular structure. The abdomen was thoroughly irrigated with sterilised saline solution and drained. Her pulse became very rapid and feeble during the operation, but improved towards the end after an enema of brandy. The drainage was maintained for a month and she steadily improved. By the middle of July the sinus had closed, and nothing abnormal could be discovered on abdominal or vaginal examination, and her general health was very good.

The origin of the peritonitis seems doubtful. No tubercles were discovered, nor was there any evidence of tubercle elsewhere. Did it start in a pyosalpinx? The pelvic viscera were so matted together, that it is impossible to say what was their condition. The peritonitis was not acute enough for appendix trouble. The result of free flushing in this case is very encouraging, but the case was not of that desperate type which we now and again experience—a form in which life is seldom prolonged for many days,—and the condition I expected to find on operation was not free pus in the general peritoneal cavity, but localised pelvic suppuration.

## Figures and Tables

**Figure f1:**
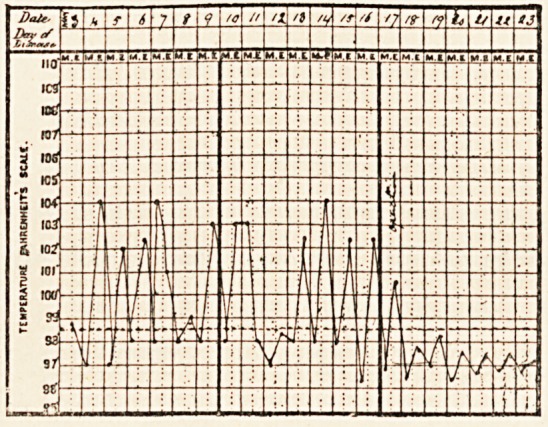


**Figure f2:**